# p53 activates G_1_ checkpoint following DNA damage by doxorubicin during transient mitotic arrest

**DOI:** 10.18632/oncotarget.3103

**Published:** 2014-12-31

**Authors:** Sun-Yi Hyun, Young-Joo Jang

**Affiliations:** ^1^ Department of Nanobiomedical Science & BK21 PLUS Global Research Center for Regenerative Medicine, Dankook University, Cheonan, Korea

**Keywords:** Mitotic DNA damage, p53, apoptosis, damage adaptation, re-replication, multiploidy

## Abstract

Recovery from DNA damage is critical for cell survival. The serious damage is not able to be repaired during checkpoint and finally induces cell death to prevent abnormal cell growth. In this study, we demonstrated that 8N-DNA contents are accumulated via re-replication during prolonged recovery period containing serious DNA damage in mitotic cells. During the incubation for recovery, a mitotic delay and initiation of an abnormal interphase without cytokinesis were detected. Whereas a failure of cytokinesis occurred in cells with no relation with p53/p21, re-replication is an anomalous phenomenon in the mitotic DNA damage response in p53/p21 negative cells. Cells with wild-type p53 are accumulated just prior to the initiation of DNA replication through a G_1_ checkpoint after mitotic DNA damage, even though p53 does not interrupt pre-RC assembly. Finally, these cells undergo cell death by apoptosis. These data suggest that p53 activates G_1_ checkpoint in response to mitotic DNA damage. Without p53, cells with mitotic DNA damage undergo re-replication leading to accumulation of damage

## INTRODUCTION

Genomic stability is necessary for the next generation to inherit perfect genetic information, but DNA damage can cause genomic instability by inducing mutations within chromosomal DNA. Cells are equipped with specific mechanisms, known as checkpoints, to protect themselves against DNA damage. During the checkpoint process, cells recognize DNA damage and stop continuous cell division until damage recovery is completed [1]. The first step of the DNA damage response involves sensor proteins such as Rad9-Rad1-Hus1, which immediately recognize the damage and recruit many transducers and effectors to the damage site [2, 3]. ATM and ATR protein kinases recruited to the damage site phosphorylate γ-H2AX as a biomarker for double-strand DNA breaks [3] in addition to phosphorylating the downstream transducers, Chk2 and Chk1 [4, 5]. Chk1 and Chk2 have been found to down-regulate Cdc25 family members, which are responsible for activating the cdk/cyclin complex [2]. This protein network finally leads to cell cycle arrest at the G_1_/S, intra-S, or G_2_/M phase through a checkpoint mechanism, and the cells are allowed plenty of time to undergo effective DNA repair. When the DNA damage cannot be repaired completely as a result of receiving high doses of the damaging agent or due to serious genetic defects, cells either progress to apoptotic death or adapt themselves to the unfavorable conditions and enter an oncogenic state [1, 5, 6]. p53 functions as a guardian of the genome by inhibiting cell growth and activating the apoptotic machinery that leads to cell death and suppresses tumors [7-9]. In particular, p53 has an essential role in the G1 checkpoint as part of the response to DNA damage [10, 11]. Cells with mutated or deleted p53 do not stop progressing through the cell cycle and can bypass the p53 checkpoint [12, 13]. p53 is regulated through phosphorylation on serine residues in a DNA damage-inducible manner by ATM/ATR and Chk1/Chk2 [14-16]. Active p53 move into the nucleus and activate the transcription of several downstream target genes including p21, which inhibits cyclin-dependent kinases (CDKs) [17]. The loss of p53 promotes tumorigenesis at a high frequency, and it is the most common genetic abnormality found in over half of all sporadic human cancers [18, 19]. In previous reports, we investigated the response to DNA damage during mitosis. DNA damage during early mitosis induces the cell to skip over the entire late mitotic process as well as cytokinesis, and instead enter a G_1_ phase with 4N-DNA contents in an ATM/Chk1-dependent manner [20, 21]. After that, multiploidy with 8N-DNA content is generated through re-replication [22]. In this report, we investigate how p53 is involved in adaption to damage resulting from a long-term response to mitotic DNA damage and connect the mitotic DNA damage response to the G_1_/S-checkpoint.

## RESULTS

### Mitotic DNA damage response in various cancer cells

**Figure 1:**
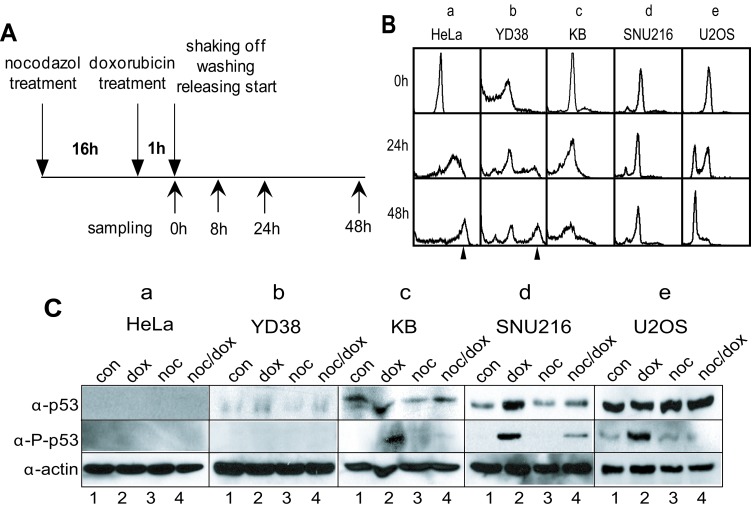
DNA damage response in various cancer cell lines (A) Experimental flowchart for mitotic DNA damage and cell harvesting. (B) DNA contents in various cancer cell lines during mitotic DNA damage response. a, HeLa; b, YD38; c, KB; d, SNU216; e, U2OS. The arrowhead indicated 8N-DNA. (C) Expression of p53 in various cancer cell lines. Activation of p53 was detected by using anti-phospho-p53(Ser15) antibody (α-P-p53). 1, unsynchronous cells (con); 2, doxorubicin treatment (dox); 3, nocodazole treatment (noc); 4, mitotic cells with doxorubicin treatment (noc/dox). Actin was detected as an estimation of total protein amounts (α-actin).

We previously reported that mitotic HeLa cells with DNA damage entered a G_1_ phase with 4N-DNA contents [20, 21] without undergoing cytokinesis, and that during damage recovery, cells with 8N-DNA contents were accumulated [22]. To examine whether or not the appearance of multiploidy is a common phenotype in the long-term response to mitotic DNA damage, we investigated the mitotic DNA damage response in various cancer cell lines including oral gingival carcinoma (YD38), tongue carcinoma (KB), stomach carcinoma (SNU216), osteosarcoma (U-2OS), and HeLa cells. The cells were synchronized at the prometaphase through treatment with nocodazole for 16 hours, and severe DNA damage was induced through treatment with 50 μM of doxorubicin for 1 hour. The mitotic cells with DNA damage were continuously cultured for 48 hours or longer after washing in fresh media to allow for DNA damage recovery (Figure 1A). Although multiploidy with 8N-DNA content were found in HeLa and YD38 cells within 24 hours of incubation (Figure 1B, a & b), this phenotype was not detected in the KB and SNU216 cells with mitotic DNA damage, even after 48 hours of damage recovery (Figure 1B, c & d). In the case of the KB cells, the number of dead cells increased during extended incubation (Figure 1B, 48h in c). Interestingly, the U-2OS cells seemed to recover and to progress to the cell cycle, even with serious DNA damage (Figure 1B, e). These results indicated that various cells cope with severe DNA damage through different responses, including becoming multiploid, stopping growth, or recovering from damage.

### p53 inhibits multiploidy formation in mitotic DNA damage response and induces apoptotic cell death in prolonged recovery period

To identify the cause for differences in the appearance of multiploidy in various cell lines, we first investigated whether or not p53 operated normally after DNA damage. Although HeLa cells are known to contain a wild-Type p53 gene, the expression of p53 is repressed by the human papilloma virus E6 [23-25]. YD38 is a p53-null cancer cell line [26], whereas KB and U-2OS had been found to be p53-positive [26-28]. To ensure consistency with these previous reports, we confirmed the absence of p53 expression in the HeLa and YD38 cell lines (Figure 1C, panels p53 & p-p53 in a & b). As expected, we confirmed p53 expression in KB, SNU216, and U-2OS (Figure 1C, panels p53 in c-e), and the p53 was positively regulated after DNA damage by phosphorylation on serine-15 (Figure 1C, lanes 2 & 4 in panels p-p53 in c-e).

**Figure 2:**
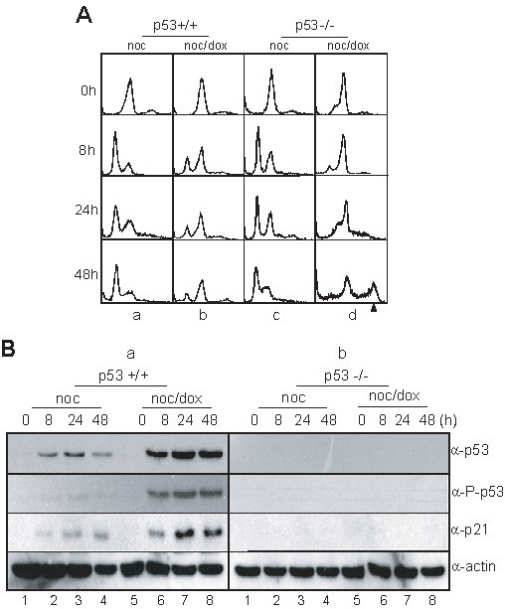
p53 involved in multiploidy formation during mitotic DNA damage response (A) DNA contents in HCT116 p53^+/+^ and p53^−/−^ cells during mitotic DNA damage response. The cell harvesting times during releasing indicated in Figure 1A. a, HCT116 p53^+/+^ treated with nocodazole; b, HCT116 p53^+/+^ with mitotic DNA damage; c, HCT116 p53^−/−^ treated with nocodazole; d, HCT116 p53^−/−^ with mitotic DNA damage. The arrowhead indicated 8N-DNA. (B) Expressions of p53 and p21 in HCT116 p53^+/+^ (a) and p53^−/−^ cells (b) during mitotic DNA damage response. Activation of p53 was detected by using anti-phospho-p53(Ser15) antibody (α-P-p53). 1-4, nocodazole treatment and releasing (noc); 5-8, mitotic cells with doxorubicin treatment and releasing (noc/dox).

To directly investigate the relationship between the formation of multiploid cells and the activation of p53 during the response to mitotic DNA damage, we examined the mitotic DNA damage response in isogenic p53^+/+^ and p53^−/−^ HCT116 cells. Both p53^+/+^ and p53^−/−^ cells in the prometaphase were released into a G1 phase during incubation without DNA damage (Figure 2A, a & c). However, prometaphasic p53^+/+^ and p53^−/−^ cells with DNA damage accumulated in a 4N-DNA stage after incubation for 24 hours (Figure 2A, 8 h & 24 h in b & d). During extended incubation for 48 hours, the p53^+/+^ cells with DNA damage were continuously arrested in a 4N-DNA stage (Figure 2A, 48 h in b), and the p53^−/−^ cells, also with DNA damage, became multiploid with 48% of cells accumulating with 8N-DNA contents (Figure 2A, 48 h in d). During prolonged incubation for recovery, the protein expression levels of p53 in the wild-type cells increased (Figure 2B, lanes 5–8 in panel α-p53 in a). Furthermore, the phosphorylation of p53 on serine-15, which is induced by DNA damage and is essential for p53 activation, was elevated in the p53^+/+^ cells (Figure 2B, lanes 5–8 in panel α-p-p53 in a). As was expected, endogenous p53 were not detected in p53^−/−^ cells with mitotic DNA damage (Figure 2B, panels α-p53 & α-p-p53 in b).

**Figure 3:**
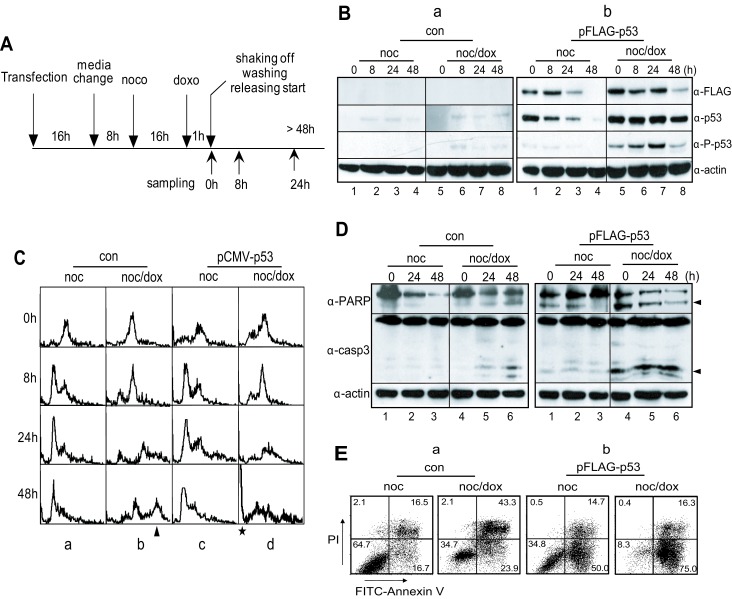
Overexpression of p53 inhibits multiploidy formation and induces apoptotic cell death in mitotic DNA damage response (A) Experimental flowchart for the ectopic expression of p53, mitotic DNA damage and cell harvesting. (B) Expression of p53 in HeLa cells with vector (a, con) and p53-expressing plasmid, pFLAG-p53 (b). Ectopic expression of p53 was detected by using anti-FLAG (α-FLAG) and anti-p53 (α-p53) antibodies, respectively. Activation of p53 was detected by using anti-phospho-p53(Ser15) antibody (α-P-p53). 1-4, nocodazole treatment and releasing (noc); 5-8, mitotic cells with doxorubicin treatment and releasing (noc/dox). (C) Accumulation of multiploidy during mitotic DNA damage response decreased by p53-expression. a, HeLa cells treated with nocodazole ; b, HeLa cells with mitotic DNA damage; c, p53-expressing HeLa cells treated with nocodazole; d, p53-expressing HeLa cells with mitotic DNA damage. The arrowhead and asterisk indicated 8N-DNA and sub-G_0_ population, respectively. (D-E) Overexpression of p53 in p53^−/−^ cells induces apoptosis severely even in early response on mitotic DNA damage. The occurrence of apoptotic cell death was observed by the cleavage of PARP (α-PARP) and caspase-3(α-casp3) (D) and by annexin V assay (E). The arrowheads in (D) indicated the active cleavage forms of PARP and caspase-3. noc, cells treated with nocodazole; noc/dox, mitotic cells with DNA damage by doxorubicin.

When p53 was ectopically expressed in HeLa cells belonging to a p53^−/−^ cell line (Figure 3A & B, lanes 1-8 in panel α-FLAG), it was activated normally through phosphorylation on serine-15 under DNA damage conditions (Figure 3B, lanes 5–8 in panel α-p-p53 in b). Both the control and the overexpressed cells with mitotic DNA damage remained in a 4N-DNA stage after incubation for 8 hours (Figure 3C, panels 6 h in b & d). Although the DNA contents increased to 8N in the control cells (Figure 3C, panels 24-48 h in b), the 8N-DNA stage did not appear in cells with overexpressed p53 during extended incubation (Figure 3C, panels 24-48 h in d). Moreover, these cells accumulated in a sub-G_0_ phase within 24 hours of recovery incubation.

To investigate the long-term response to mitotic DNA damage, HeLa cells arrested in prometaphase were treated with doxorubicin to induce DNA damage and were released into fresh media for 48 hours to recover from the damage. Active cleavage of caspase-3 and PARP was weakly detected 48 hours after release (Figure 3D, lane 6 in the upper & middle panels in a). Under this condition, 43.3% of the cells were double-positive for PI and annexin V, 34.7% were double-negative for PI and annexin V, and 23.9% were annexin V-positive (Figure 3E, noc/dox in a). The data suggest that it was not until the cells were incubated for 48 hours that more than 65% became defective, and the percentage of apoptotic cells were no more than 24% of the total. p53 was ectopically expressed in the HeLa cells and the mitotic DNA damage response of these cells was analyzed under the same conditions. Active cleavage of caspase-3 and PARP was clearly detected just after release (Figure 3D, lanes 4-6 in upper & middle panels in b). Moreover, only 16.3% of the cells were double-positive for PI and annexin V, and 75.0 % of the cells were annexin V positive. As expected, more than 90% of the p53 overexpressed cells with mitotic DNA damage were defective (Figure 3E, noc/dox in b), indicating that p53 induced apoptosis in mitotic cells with severe DNA damage while inhibiting damage adaptation.

**Figure 4:**
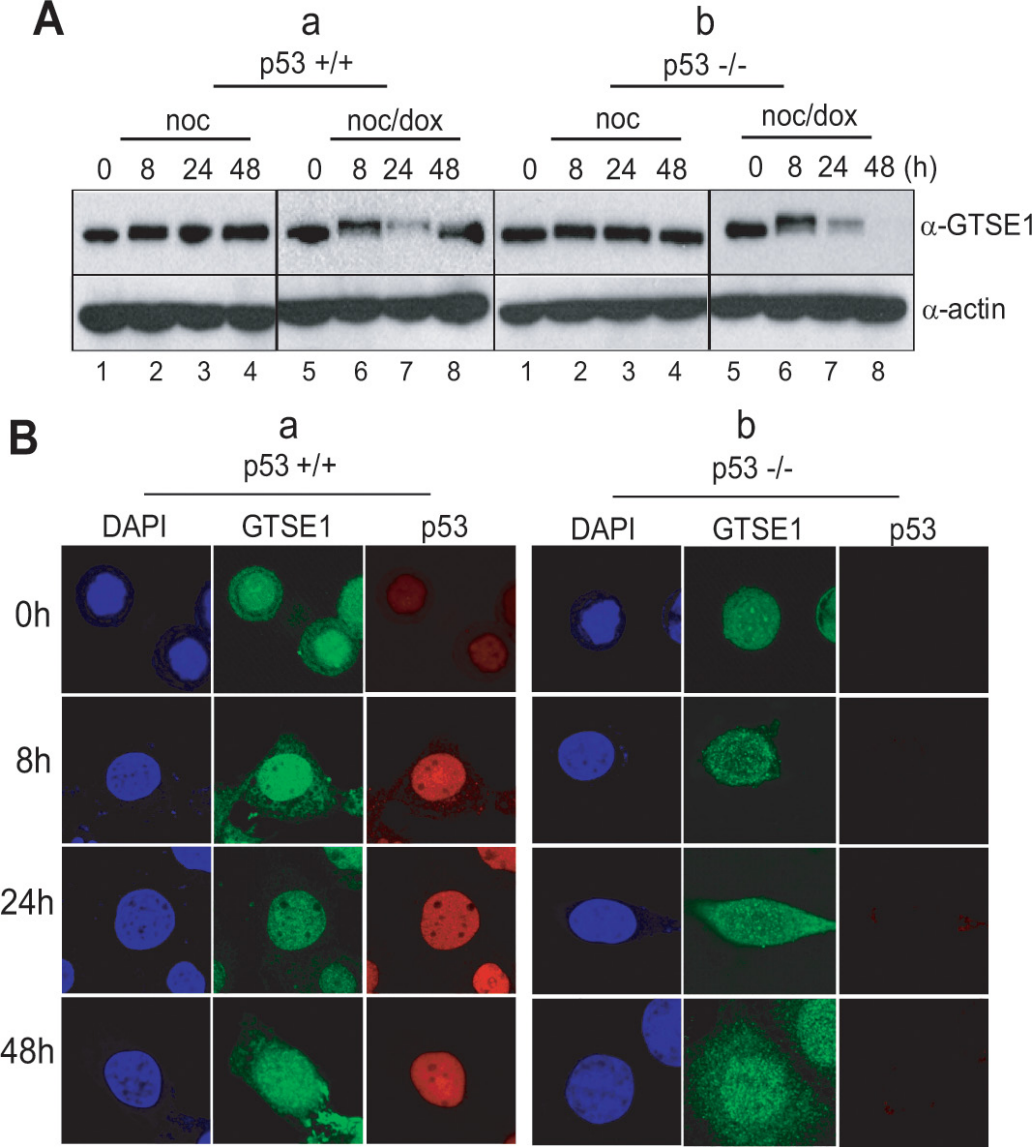
p53 and GTSE1 function reciprocally in mitotic DNA damage response (A) Expression of GTSE 1 in p53^+/+^ (a) and p53^−/−^ cells (b) during releasing from mitotic DNA damage for 48 hours. 1-4, nocodazole treatment and releasing (B) Subcellular localization of GTSE-1 during releasing in HCT116 p53^+/+^ (a) and p53^−/−^ cells (b).

The GTSE-1 protein is known to be a negative regulator of p53. With respect to the G1 checkpoint and the recovery period, it is normally expressed during the G_2_ and the S phase [29, 30], and suppresses apoptosis for normal cell growth. Indeed, when mitotic cells without DNA damage undertook normal cell division, GTSE-1 was highly expressed during extended culture (Figure 4A, lanes 1–4 in upper panels in a & b). The level of p53 decreased and was inactivated under this condition (Figure 3B, lanes 1-4 in a). However, when p53^+/+^ cells were incubated for 48 hours to recover from mitotic DNA damage, p53 had been activated (Figure 2B, lanes 5-8 in a) and GTSE-1 expression also remained at a high level during incubation (Figure 4A, lanes 5–8 in upper panels in a). Conversely, in the p53^−/−^ cells, GTSE-1 decreased dramatically during extended incubation (Figure 4A, lanes 5–8 in upper panels in b). Moreover, GTSE-1 and p53 were co-localized in a nuclear region during mitotic DNA damage recovery for 24-48 hours (Figure 4B, a). Conversely, GTSE-1 did not accumulate in the nucleus in the absence of p53 within 24-48 hours after release (Figure 4B, b). These results suggest that p53 may be restrained by GTSE-1 during early damage recovery within 8 hours, and that cells attempt to recover during the checkpoint. When cells were exposed to severe damage stress, the level of GTSE-1 expression remained constant and p53 became active. Eventually, the cells appear to abandon recovery attempts and are removed through apoptosis. In the p53^−/−^ cells, GTSE-1 expression was defunct and/or was not localized in the nucleus. Therefore the cells ceased DNA replication for damage adaptation.

### p53 does not affect cytokinesis failure and pre-RC assembly in mitotic DNA damage recovery

**Figure 5:**
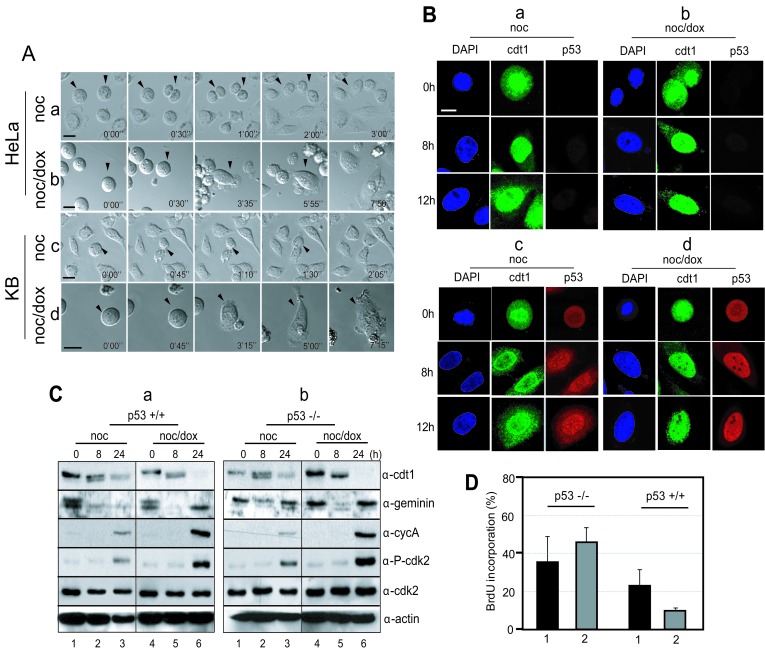
p53 blocked DNA replication during mitotic DNA damage response (A) Cellular phenotype during mitotic DNA damage response under time lapse microscopy. a, mitotic p53^−/−^ cells; b, mitotic p53^−/−^ cells with DNA damage; c, mitotic p53^+/+^ cells; d, mitotic p53^+/+^ cells with mitotic DNA damage. p53 does not have influence on the cytokinesis failure, which was a feature of mitotic DNA damage response within 8 hours. (B) Subcellular localization of cdt1 and p53. For investigation of pre-RC assembly, we observed nuclear localization of cdt1, which is a component of pre-RC complex during releasing for 12 hours. a, mitotic p53^−/−^ cells; b, mitotic p53^−/−^ cells with DNA damage; c, mitotic p53^+/+^ cells; d, mitotic p53^+/+^ cells with mitotic DNA damage. (C) Molecular changes during mitotic DNA damage response. Mitotic p53^+/+^ (a) and p53^−/−^ (b) cells were released into fresh media and harvested at indicated time point. 1-3, mitotic cells without DNA damage (noc); 4-5, mitotic cells with DNA damage by doxorubicin treatment (noc/dox). The indicated proteins were detected by using anti-cdt1 (α-cdt1), anti-gemenin (α-geminin), anti-cyclin A (α-cycA), anti-phosph-cdk2(Thr160) (α-P-cdk2), anti-cdk2 (α-cdk2), and anti-actin (α-actin) antibodies. (D) Investigation of DNA replication during mitotic DNA damage response. p53^−/−^ and p53^+/+^ cells were cultured on the cover glass with BrdU, and cells incorporated with BrdU were counted. 1, Mitotic cells released for 24 hours; 2, Mitotic cells with DNA damage released for 24 hours.

To investigate whether p53 is involved in cytokinesis failure in the short-term response to mitotic DNA damage [20-22], both p53^−/−^ (Figure 5A, a & b) and p53^+/+^ cells (Figure 5A, c & d) synchronized at prometaphase were observed under a live cell imaging microscope. Normally, in both cases, prometaphasic cells without damage perform late mitotic events and divide into two daughter cells within 1 hour of release, suggesting that cell division occurs regardless of the presence of p53 (Figure 5A, a & c). The p53-negative cells with mitotic DNA damage failed to perform cytokinesis even after 3 hours of incubation in fresh medium (Figure 5A, b). Under the same conditions, the p53-positive cells were found to have the same phenotype as the p53-negative cells (Figure 5A, d). These data suggest that the existence and activation of p53 does not affect cytokinesis failure or the induction of 4N-DNA G_1_ phase during short-term recovery.

To initiate replication of the eukaryotic genome, pre-RC (pre-replicative complex) assembly is an essential event, which begins in late mitosis and continues to the G_1_ phase. The origin recognition complex (ORC) is a sequence-specific DNA binding protein complex and is recognized as the primary origin for pre-RC assembly. Cdt1 and Cdc6 are localized on ORC to promote the firing of the origin [31, 32]. To determine whether or not pre-RC assembly preceded the re-replication following cytokinesis failure in the mitotic DNA damage response, and whether or not this assembly occurred in both p53^+/+^ and p53^−/−^ cells, the localization of Cdt1was detected in cells using confocal microscopy (Figure 5B). In p53^−/−^ cells, Cdt1 was localized in the nucleus during incubation after nocodazole arrest and slowly diffused into the cytoplasm after release for 12 hours (Figure 5B, *Cdt1* in a). Although the localization of Cdt1 in the nucleus was also detected in these cells with mitotic DNA damage, Cdt1 continued to accumulate in the nucleus even after 12 hours (Figure 5B, *Cdt1* in b). In p53^+/+^ cells, Cdt1 was also localized in the nucleus and its diffusion into the cytoplasm was detected in cells 8 hours after release (Figure 5B, *Cdt1* in c). The Cdt1 in the p53^+/+^ cells with mitotic DNA damage was localized tightly in the nucleus during incubation in fresh media in a pattern similar to those in p53^−/−^ cells with mitotic DNA damage (Figure 5B, *Cdt1* in b & d). Interestingly, the localization pattern for p53 was different depending on the mitotic DNA damage in the cells. p53 in cells without DNA damage was not localized tightly in the nucleus during the cell cycle progression (Figure 5B, *p53* in c), but cells with mitotic DNA damage retained p53 localization in the nucleus even after 12 hours of incubation (Figure 5B, *p53* in d). These data indicate that the nuclear localization of Cdt1 for pre-RC formation was not relevant to the presence of p53 during the mitotic DNA damage response. Geminin, a Cdt1 inhibitor also disappeared for pre-RC formation from mitotic DNA damage in both p53^−/−^ and p53^+/+^ cells after 8 hour-release (Figure 5C, lanes 5 in α-geminin in a & b). Additionally, the inactivation of Cdk2 was detected at the same time for both types of cells (Figure 5C, lanes 5 in α-p-cdk2 in a & b), and the active phosphorylation of cdk2 on threonine-160 as well as the level of cyclin A, the partner of Cdk2 during the S phase, were restored within 24 hours of release (Figure 5C, lanes 6 in α-cycA & α-p-cdk2 in a & b). A BrdU incorporation assay revealed that p53^−/−^ cells perform DNA replication after 24 hours of release in response to mitotic DNA damage (Figure 5D, lane 2 in p53^−/−^). Conversely, the ratio of the BrdU incorporation was remarkably low in p53^+/+^ cells with mitotic DNA damage (Figure 5D, lane 2 in p53^+/+^), suggesting that DNA replication in p53^+/+^ cells is blocked after pre-RC formation during mitotic DNA damage recovery. These data indicated that pre-RC is formed in both types of cells with mitotic DNA damage, and that cells seem to enter into the S phase normally. However, DNA replication might be inhibited by p53, which was tightly localized in the nucleus during release after mitotic DNA damage (Figure 5B, panels p53 in d and Figure 5D, graph 2 in p53^+/+^).

### p21 inhibits DNA replication during mitotic DNA damage recovery of p53^+/+^ cells

During DNA damage recovery, the prometaphasic cells accumulated in the interphase without undergoing cytokinesis and formed pre-RC within 8 hours of incubation, regardless of the presence of p53 (Figure 5B, b & d and Figure 5C, lanes 5 in α-cdt1 in a & b). During extended incubation, both types of cells moved into the S-phase, where cdt1 disappeared and Cdk2/cyclinA was activated by phosphorylation (Figure 5C, lanes 6 in α-cdt1, α-cycA, and α-p-cdk2 in a & b). In spite of these similar phenotypes for both types of cells during the mitotic DNA damage response, multiploidy was detected only in p53^−/−^ cells (Figure 1B, a & b and Figure 2A, d). To understand the formation of multiploidy during mitotic DNA damage recovery in p53^−/−^ cells, we investigated the relevance of p21, one of the p53 downstream targets and a Cdk2 inhibitor. When DNA damage was induced in mitotic p53^+/+^ cells, the endogenous level of p21 dramatically increased during extended release in the same pattern as p53 expression (Figure 2B, lanes 5-8 in a).

**Figure 6:**
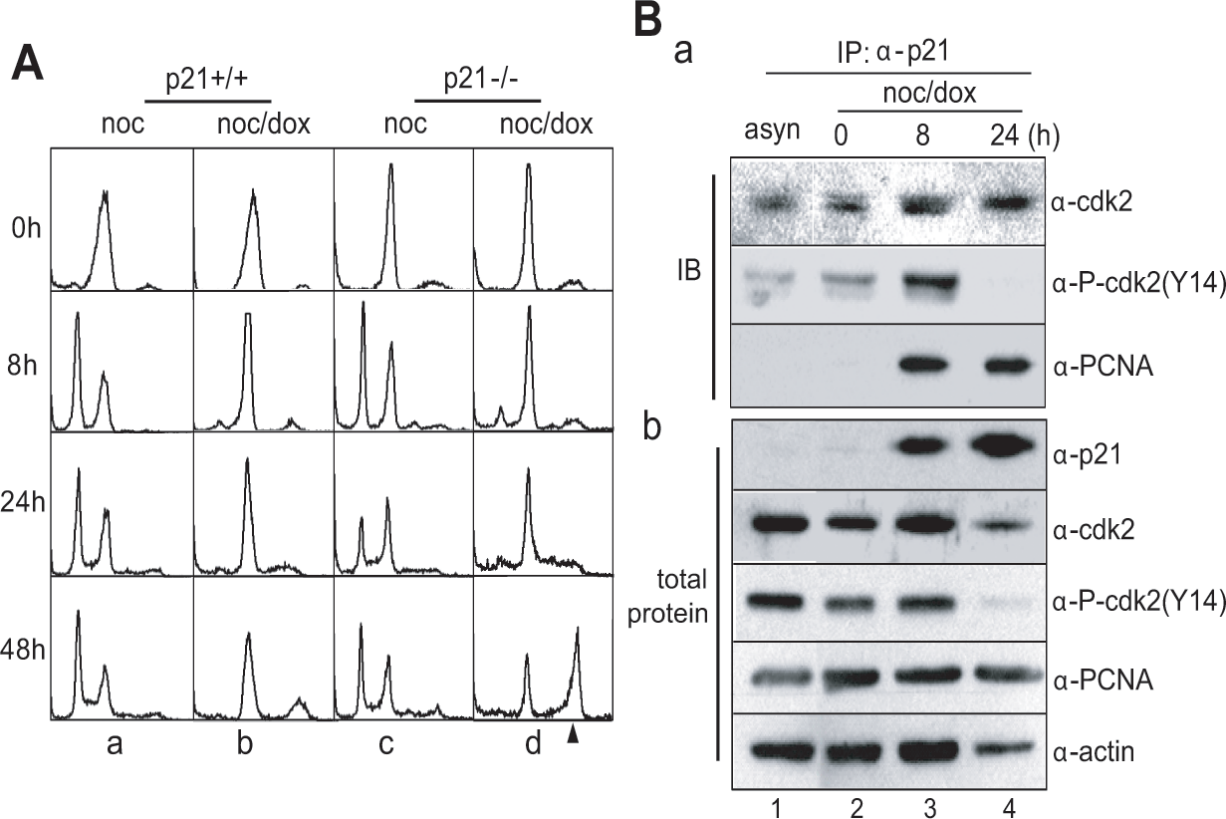
p21 blocked DNA replication in mitotic DNA damage response (A) DNA contents in HCT116 p21^+/+^ and p21^−/−^ cells during mitotic DNA damage response. The 8N-DNA contents were accumulated in HCT116 p21^−/−^ cells during mitotic DNA damage response. The cell harvesting times during releasing indicated in Figure 1A. a, HCT116 p21^+/+^ treated with nocodazole; b, HCT116 p21^+/+^ with mitotic DNA damage; c, HCT116 p21^−/−^ treated with nocodazole; d, HCT116 p21^−/−^ with mitotic DNA damage. The arrowhead indicated 8N-DNA. (B) Interaction between p21 and Cdk2 or PCNA during mitotic DNA damage response. (a) Endogenous p21 in mitotic cells with DNA damage was immunoprecipitated (IP), and bound cdk2 and PCNA was detected by immunoblot (IB). The amount of each protein in cell extracts was indicated in (b). 1, immunoprecipitation of p21 in asynchronous cells (asyn); 2-4, immunoprecipitation of p21 in mitotic cells with DNA damage (noc/dox) during releasing for indicated time.

Without DNA damage, both p21^+/+^ and 21^−/−^ cells arrested in the prometaphase progressed through the normal cell division cycle within 8 hours of incubation in a manner independent of the presence of p21 (Figure 6A, a & c). However, mitotic p21^+/+^ cells with DNA damage did not replicate their DNA and were arrested in a 4N DNA stage (Figure 6A, b). When mitotic p21^−/−^ cells were treated with doxorubicin and released into fresh media, cells with 8N-DNA content accumulated during extended incubation of 48 hours (Figure 6A, d).

At the molecular level, endogenous p21 protein interacted with both Cdk2 and Cdk2 phosphorylated on Tyr-14 (Figure 6B, α-cdk2 & α-P-cdk2(Y14) in a). Since cells accumulated in the G_1_-S phase after 24 hours of incubation, Cdk2 likely became active, resulting in removal of the inhibitory phosphorylation on Tyr-15 (Figure 6B, lane 4 in α-P-cdk2(Y14) in b). Therefore, the interaction between p21 and Cdk2 would not be detected (Figure 6B, lane 4 in α-P-cdk2(Y14) in a). Furthermore, p21 interacted with the proliferating cell nuclear antigen (PCNA) 8 hours after release (Figure 6B, lanes 3-4 in α-PCNA in a), suggesting that when p21 is induced by p53, DNA replication might be inhibited in the S phase through an interaction between Cdk2 and PCNA during the mitotic DNA damage response.

## DISCUSSION

DNA damage frequently occurs as a result of factors endogenous and exogenous to the cells and can induce cell death or tumorigenesis. Depending on the intensity of the damage, cells can recover from damage, adapt to the damage, or be removed due to death. In previous reports, we studied the response to DNA damage that occurred in the prometaphase, rather than the interphase. DNA damage caused by doxorubicin shock and gamma-irradiation in mitotic cells did not induce mitotic arrest during recovery, and these cells bypassed late mitotic events including cytokinesis [20, 21]. Moreover, cells with 4N-DNA contents entered the G_1_-phase within 8 hours of recovery incubation, even though the DNA breaks were still present.

Previously, it was reported that prolonged mitosis by treatment with nocodazole for 24-36 hours lead cell death or mitotic slippage, and that G_1_-like arrest occur by p53-dependent manner under low concentration of mitotic inhibitor [33, 34]. In this report, we focused on the long-term recovery response to mitotic DNA damage. For this, cells were treated with nocodazole for 12-16 hours for collection of mitotic cells. These cells were divided and reentered into G_1_ phase within 30 min in fresh medium (Figure 5A). Cell death or mitotic slippage was not detected. Under the same condition, mitotic cells with DNA damage were arrested in G_1_-like phase and induced cell death in p53-dependent way. Indeed, mitotic cells collected from cell culture which was released from thymidine double block also responded on the DNA damage in the same phenotypes (Supplementary data-1), suggested that cell death or mitotic slippage and re-replication during prolonged recovery of mitotic DNA damage are not the effects from prolong mitosis.

**Figure 7:**
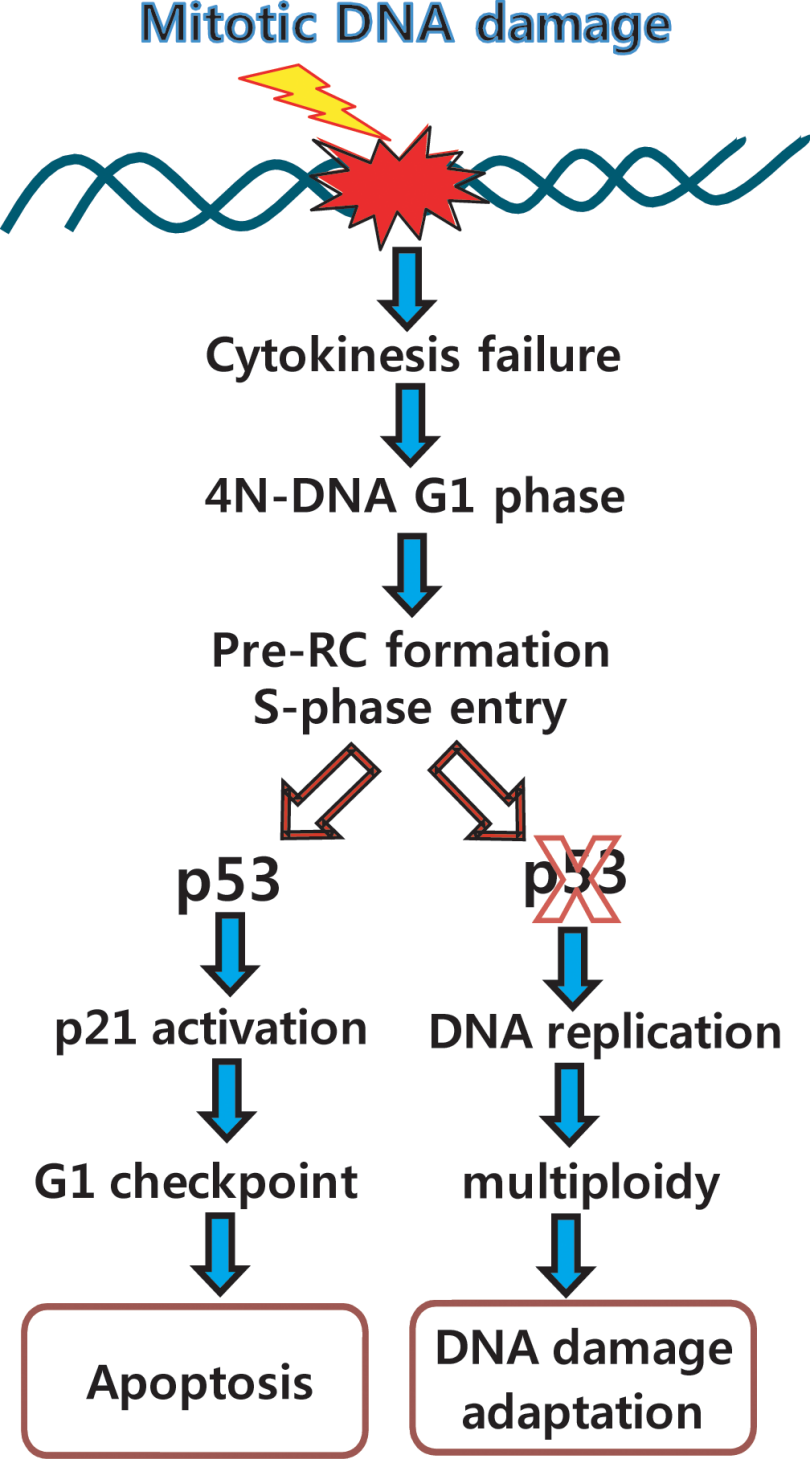
Overview of mitotic DNA damage response: connection between mitotic DNA damage and G1-S checkpoint by p53 When DNA damage stresses occur in middle of the mitosis, ATM-Chk1 pathway is activated and Plk1 is dephosphorylated by PP2A and other phosphatases within 6 hours from release into fresh media [20, 21]. Then, cells fail to finish-up cytokinesis, progress into interphase with 4N-DNA contents, and initiate S-phase by pre-RC formation. Although normal cells are ready to initiate DNA replication with pre-RC assembly and activation of Cdk2 under the severe DNA damage condition, these cells are blocked in DNA replication due to activation of G_1_-S checkpoint, and finally are removed by induction of apoptosis within 24h. However, in the case of p53-inactive or null cells, although DNA damage remains in cells, they go through G_1_ phase with damage, and undergo re-replication within 24 hours of incubation for recovery. Since then, cells might be adapted in multiploidy, and destined to be a cancer.

In p53^−/−^ cells, multiploidy with 8N-DNA content was detected after 24-48 hours of damage recovery (Figure 1B, a & b and Figure 2A, d). Conversely, multiploidy was not induced in cells with p53^+/+^ cells (Figure 1B, c-e and 2A, b). Moreover, the ectopic expression of p53 reduced the population of cells with 8N-DNA contents (Figure 3C, panels b), indicating that p53 suppresses multiploidy induction during long-term damage adaptation. Since 8N-DNA formation is caused by DNA replication, we investigated the involvement of p53 in pre-RC formation in the initiation of the S-phase. Cdt1 is a major component of pre-RC and is normally assembled on the nucleus in both p53^−/−^ and p53^+/+^ cells with the same amount of mitotic DNA damage (Figure 5B, b & d). Cdt1 becomes inactivated and is released from the nucleus during the S phase by Cdk2 activation [31, 35, 36], and geminin binds directly to Cdt1 to inhibit the formation of pre-RC [37, 38]. As expected, the activation of geminin and Cdk2 is reduced in both p53^+/+^ and p53^−/−^ cells during nuclear localization of Cdt1 (Figure 5B, b & d and 5C, panels α-Cdt1, α-CycA, & α-p-Cdk2). Under this condition, Cyclin A, a Cdk2 partner in initiating the S-phase, is not expressed (Figure 5C, lanes 4-5 in a & b). These data indicate that p53 is not involved in the suppression of the pre-RC assembly to inhibit DNA replication. On the other hand, BrdU incorporation dramatically decreased in p53-positive cells (Figure 5D, graphs 2). The inhibition of DNA replication by p53 might be mediated by p21, one of the inhibitors of Cdks. p21 interacts with Cdk2 and PCNA and simultaneously inhibits the progression of the S-phase and DNA replication (Figure 6B). Based on these data, p53 might be involved in the inhibition of the DNA replication process and has no effect on the events that occur before the initiation of the S phase. Both p53^−/−^ and p53^+/+^ cells failed to complete the late events of mitosis and did not undergo cytokinesis (Figure 5A, b & d), indicating that p53 has no influence in late mitotic progression, including cytokinesis, in the short-term response to mitotic DNA damage. So far, p53 was observed to be involved in the G_1_ phase checkpoint, which protects against an abnormal genome and induces cell cycle arrest before the S phase in response to DNA damage [39-42]. Daughter cells that divided during protracted mitosis are eventually arrested at the G_1_ phase in a p53-dependent manner [43]. In our study, p53-positive cells with mitotic DNA damage did not progress to the DNA replication step, in contrast to p53^−/−^ cells with mitotic DNA damage. These data indicate that the G_1_ checkpoint is activated in response to mitotic DNA damage in the presence of p53, and that the mitotic DNA damage response is connected to the G_1_ checkpoint by p53. If cells continue to possess damaged DNA, apoptosis is induced in a p53-dependent manner. Actually, the sub-G_0_ population of cells over-expressing p53 with mitotic DNA damage increased even within 8 hours of incubation (Figure 3C,). The active cleavage of caspase-3 and PARP also increased within 8 hours in cells expressing p53 compared to p53^−/−^ cells (Figure 3D & E, b). On the other hand, cell viability increased with mitotic DNA damage when p53 was inactive or depleted (Figure 3D & E, a). Instead of an increase in viability, cells become multiploid through the accumulation of 8N-DNA contents during re-replication, indicating adaption to the DNA damage. In conclusion, we have demonstrated that the mitotic DNA damage response is connected to the p53-mediated G_1_ checkpoint for damage recovery. The model in Figure 7 suggests that in the short-term response, mitotic cells with DNA damage skip the late mitotic processes. In the long-term response, cells choose their fates: recovery, death, or adaptation. Under this condition, cell death or damaged cell adaptation is determined by the presence of p53. When p53 is not expressed and is not activated in the cells, mitotic DNA damage induces the accumulation of 8N-DNA contents, and the cells might become tumorigenic. Conversely, p53 induces a G_1_ checkpoint mediated by p21 in the mitotic DNA damage response, and cells are blocked from replicating DNA. These cells are removed through apoptosis in a short period of time.

## MATERIALS AND METHODS

### Cell culture, treatments and transfection

Various cancer cells were maintained in DMEM containing 10% FBS (Hyclone). To synchronize in prometaphase, cells were treated with nocodazole (100 ng/ml, Sigma) for 16 hours and collected by shake-off. For induction of DNA damage, mitotic cells were treated with doxorubicin (5 μM, Sigma) for 1h. For ectopic expression, cells were transfected as described previously with modification [21]. Briefly, cells were incubated in DMEM containing 5% FBS before 3 hours of transfection, and added with the precipitates of plasmid DNA and calcium salt. After 16 hours, cells were washed, and harvested for further study after incubation for 24 hours.

### Flow cytometry and Annexin V assay

For analysis of DNA contents, cells were trypsinized, fixed in 80 % ethanol for 16 hours, and treated with RNaseA (100 μg/ml) at 37 °C for 2 hours. Cells stained with propidium iodide (40 μg/ml) were analyzed by flow cytometry with 30,000 events (FACScaliber, Becton Dickinson). For analysis of cell death, we followed the manufacture's manual of Annexin V-FITC apoptosis analysis kit (BD Pharmingen). Briefly, cells were trypsinized and washed by ice-cold PBS twice. 1 × 10^5^ cells are suspended in 100 μl of Binding buffer, and 5 μl of Annexin V-FITC (BD Pharmingen) and propidium iodide were added. After incubation for 15 min, 400 μl of Binding buffer were added, and analyses were carried out using a flow cytometry. Data were processed with WinMDI software.

### Western Blotting and antibodies

Cells were lysed in NP-40 cell lysis buffer [21], and lysates were separated on SDS-PAGE. Proteins were transferred to a PVDF, and the primary and secondary antibodies were incubated in TBST containing 5% skim-milk. Protein signals were visualized using the ECL^TM^ system (Amersham Biosciences). Antibodies for p53, phospho-p53(S15), phospho-Cdk2(T160), Cdk2, PCNA and Caspase-3 were obtained from Cell Signaling. Antibodies for geminin, cdt1, cyclin-A, and PARP were obtained from Santa Cruz Biotechnology. Anti-p21, phospho-Cdk2(T14), and GTSE1 antibodies were purchased from Abcam. Anti-FLAG antibody was obtained from Agilent.

### Confocal Microscopic analysis and live cell imaging

For confocal microscopic analysis, cells were cultured on coverslips and fixed in 4% paraformaldehyde, followed by treatment with cold methanol. Cells were incubated with the primary antibody and FITC or Cy3 labeled anti-IgG antibody (Jackson ImmunoResearch Inc.). The fluorescence signals were detected and captured by confocal microscopy (LSM510). For observation of live cells, mitotic cells with DNA damage were grown on the glass bottom dish during detection by microscope (Zeiss Axiovert S100) with a LCI long-term microincubator chamber system with CO2.

### BrdU incorporation

Cells cultured on coverslips were treated with bromodeoxyuridine (10 μM, Sigma) during incubation, and were fixed in 4% paraformaldehyde for 15 min. After washing, cells were incubated in 0.3% Triton X-100 for 15 min. Cells were incubated in 2 M HCl for 20 min, were neutralized using 0.1 M sodium borate for 2 min, and were stained with FITC conjugated BrdU antibodies (BD Pharmingen) in PBS containing 10% Tween 20 and 2% horse serum. The fluorescence signals were detected and captured by confocal microscopy (LSM510).
